# Serial echocardiography–based diagnosis of a left atrial thrombus mimicking myxoma in severe mitral regurgitation

**DOI:** 10.1093/ehjcr/ytag282

**Published:** 2026-04-22

**Authors:** Daiki Mori, Sakiko Nunotani, Hitoshi Takahashi, Atsushi Hiraide

**Affiliations:** Department of Emergency Medicine, Nagayama Hospital, 1-11-10, Okubo-higashi, Kumatoricho, Sennangun, Osaka 590-0406, Japan; Rinku General Medical Center, Senshu Trauma and Critical Care Center, Rinku Orai-kita 2-23, Izumisano City, Osaka 598-8577, Japan; Department of Emergency Medicine, Nagayama Hospital, 1-11-10, Okubo-higashi, Kumatoricho, Sennangun, Osaka 590-0406, Japan; Department of Emergency Medicine, Nagayama Hospital, 1-11-10, Okubo-higashi, Kumatoricho, Sennangun, Osaka 590-0406, Japan; Department of Emergency Medical Sciences, Meiji University of Integrative Medicine, Hiyoshi-cho, Nantan-shi, Kyoto 629-0392, Japan

## Case description

**Figure 1:**
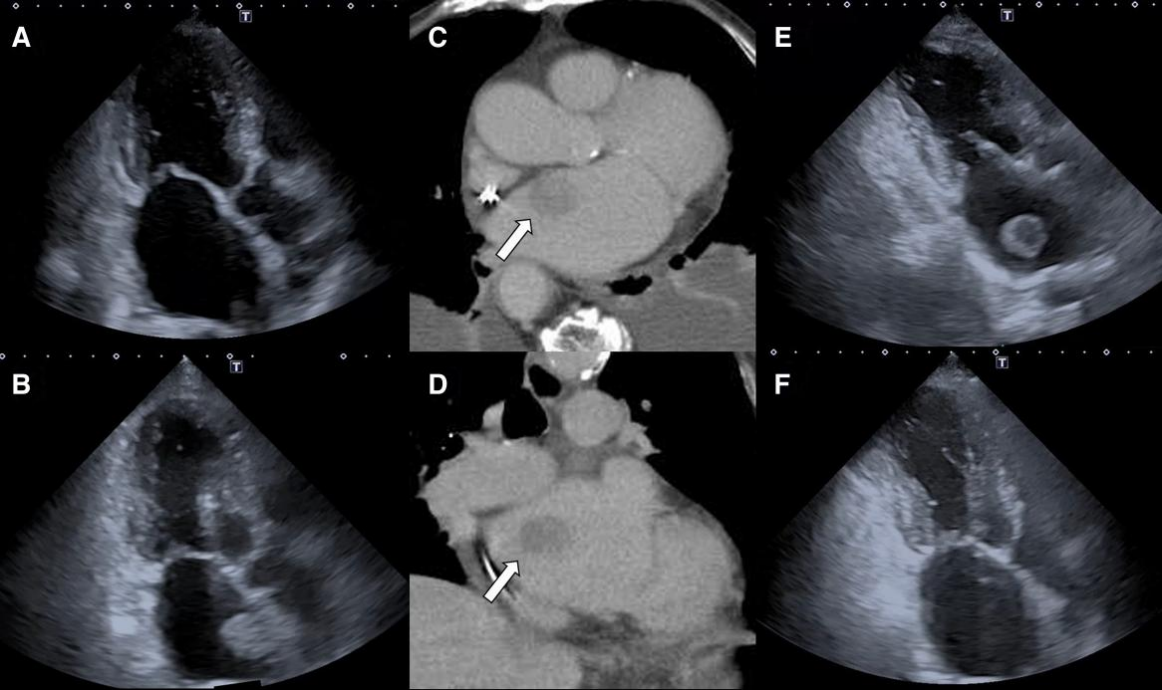
Serial transthoracic echocardiography and computed tomography findings. (*A*) No left atrial mass was observed 26 days before admission. (*B*) A mobile mass measuring 25.0 × 23.9 mm was detected in the left atrium on day 1. (*C* and *D*) Contrast-enhanced computed tomography demonstrated an intracardiac mass (white arrow). (*E*) On day 5, the mass was free-floating in the left atrium and showed morphological changes compared with day 1. (*F*) The mass disappeared from the left atrium by day 6.

A 94-year-old woman was admitted with bowel obstruction. Transthoracic echocardiography (TTE) performed 26 days earlier showed severe mitral and aortic regurgitation but no left atrial mass (*Figure 1A*; Supplementary material online, *Video S1*).

On admission, TTE revealed a mobile, smooth-surfaced mass measuring 25.0 × 23.9 mm attached to the interatrial septum (*Figure 1B*; Supplementary material online, *Video S2*).

Contrast-enhanced computed tomography demonstrated an intracardiac mass similar in size to that on TTE (*Figure 1C* and *D*). No findings suggestive of malignancy were identified, making metastatic cardiac tumour unlikely. Definitive diagnosis by histological examination or advanced imaging was not feasible because the patient declined invasive investigations and treatment. Anticoagulation was not initiated based on patient preference.

On day 5, she developed acute neurological deficits, and cerebral infarction was confirmed by computed tomography. TTE demonstrated that the mass had become free-floating within the left atrium and had developed layered echocardiographic features (*Figure 1E*; Supplementary material online, *Video S3*). Based on the rapid appearance of the mass and the morphological changes on serial TTE, the lesion was clinically diagnosed as a thrombus. On day 6, TTE confirmed complete disappearance of the mass (*Figure 1F*; Supplementary material online, *Video S4*), and she died from a presumed thromboembolic cerebral infarction.

Differentiation between left atrial thrombus and myxoma may be challenging on TTE.^1^ Left atrial thrombus is uncommon in severe mitral regurgitation.^2^ In this case, a thrombus attached to the interatrial septum mimicked a myxoma despite severe MR. Further evaluation and appropriate intervention should be considered.^1,3^ In elderly patients, however, invasive intervention may be declined, necessitating clinical decision-making based on limited information. Repeated TTE provided diagnostic information and was useful for explaining the risk of thromboembolism to the patient. This case illustrates the importance of using TTE with an understanding of its role and limitations.

## Supplementary Material

ytag282_Supplementary_Data

## Data Availability

The data underlying this article are available in the article and its online Supplementary material.
