# Supplementary material for: Serial echocardiography–based diagnosis of a left atrial thrombus mimicking myxoma in severe mitral regurgitation
Source: Eur Heart J Case Rep. 2026 Apr 22;10(5):ytag282. doi: 10.1093/ehjcr/ytag282 (PMC13158966; doi:10.1093/ehjcr/ytag282)
Supplement: ytag282_Supplementary_Data [file ytag282_supplementary_data.zip › Supplementary Data captions.docx]

**Supplementary Data**

Video S1. No apparent mass is observed in the left atrium 26 days earlier.

Video S2. A 25.0 × 23.9 mm mass was detected in the left atrium on day 1.

Video S3. On day 5, the mass was floating in the left atrium and showed morphological changes compared to those on day 1.

Video S4. The mass disappeared from the left atrium by day 6.
